# The promise and peril of data-driven approaches: interpreting the association of insomnia, sleep apnea, and COMISA with mortality in US veterans

**DOI:** 10.1007/s44470-026-00093-9

**Published:** 2026-04-27

**Authors:** Ripu D. Jindal

**Affiliations:** Birmingham VA Medical Center, 700 19th Street South, Birmingham, AL 35233 USA

Capitalizing on the electronic medical records from Veterans Health Administration (VHA), the largest integrated healthcare system in the United States, Razjouyan and colleagues [1] performed a retrospective analysis in a cohort of 1,720,090 patients to examine all-cause mortality rates in insomnia, sleep apnea, and comorbid insomnia and sleep apnea (COMISA) and found that insomnia was a stronger predictor of mortality than either sleep apnea alone or COMISA. The findings are provocative and stand in contrast to much of the existing literature. Whether this result reflects a true and underrecognized phenomenon or instead serves as a cautionary example of the limits of a particular data-driven approach, the report is both noteworthy and deserving of attention.

In clinical trials of comorbid insomnia and sleep apnea, combined treatment with cognitive behavioral therapy for insomnia (CBT-I) and positive airway pressure (PAP) for obstructive sleep apnea has consistently improved insomnia symptoms, while improvements in PAP adherence have been less consistent [2–4]. Conversely, PAP treatment alone may also alleviate insomnia symptoms in some patients [5]. Taken together, these findings have led most experts to endorse an integrated treatment approach that addresses both disorders simultaneously. In practice, however, limitations in resources and patient preferences often require clinicians to prioritize one condition over the other. If replicated and confirmed, the findings reported by Razjouyan and colleagues could support prioritizing the screening and treatment of insomnia over that of sleep apnea. Such a shift could also carry important implications for public policy and the allocation of healthcare resources.

The report from Razjouyan and colleagues leverages electronic medical records and includes multiple alternative explanations for the findings. The inherent imprecision in analyzing data from medical records seems especially true for the diagnoses of insomnia and sleep apnea. Multiple population studies have investigated the relationship between sleep metrics and mortality. In the UK Biobank cohort, sleep regularity was a stronger predictor of all-cause mortality than sleep duration [6]. In the *Multi-Ethnic Study of Atherosclerosis*, a composite sleep score composed of 13 sleep metrics predicted mortality [7], with night-to-night sleep regularity, total sleep time, and the Apnea–Hypopnea Index as the largest drivers of the association.

Multiple studies suggest that the associations between sleep disorders and mortality are not uniform but instead depend on specific phenotypic and biological features within insomnia and sleep apnea. In obstructive sleep apnea, for example, hypoxic burden predicted cardiovascular mortality in pooled analyses from the *Outcomes of Sleep Disorders in Older Men Study* and the *Sleep Heart Health Study* [8]. Similarly, an excessively sleepy subtype of obstructive sleep apnea has been associated with particularly elevated cardiovascular risk [9]. More recently, a “gene by excessive daytime sleepiness” analysis in patients with sleep apnea identified genetic loci linked to cardiovascular risk, insulin resistance, thiamine deficiency, and resveratrol-related mechanisms [10], offering further insight into the biological pathways that may underlie risk in this subtype. Heterogeneity is also evident in insomnia. Not all cases of insomnia are likely to confer the same cardiovascular risk; for example, the phenotype of insomnia accompanied by objectively short sleep duration has been associated with greater incidence of cardiovascular disease and mortality in the *Sleep Heart Health Study* [11] and *Penn State Cohort* [12], respectively.

The intrinsic risk of decontextualization in a data-driven approach is particularly relevant to the clinical practice of medicine. As clinicians, we know that the pre-test probability of a disorder has a bearing on the risk of false-positive and false-negative results on diagnostic tests [13]. Similarly, the lack of an explicit a priori hypothesis increases the risk of spurious findings [14] and can point us in the wrong direction. A related concern stems from what can be called data fatigue. Given the unprecedented pace at which biomedical research is generating data, we run the risk of a saturation effect, in which patients, providers, and policymakers start to pick and choose and lose perspective on what is most important. The proverbial baby may be thrown out with the bath water; or worse, the baby is thrown out, and the bath water is retained. The efficiency and “intellectual humility” of a data-driven approach must be weighed against its greater susceptibility to spurious findings and data fatigue. In that balance, the decontextualization inherent in a data-driven approach is a double-edged sword: Stripped of context, a finding is more vulnerable to misinterpretation, yet that same distance from prior assumptions may also open the door to new and potentially useful insights.

If we can remain mindful of the pitfalls, data-driven approaches can usefully inform the design of more definitive studies, such as Mendelian randomization studies and randomized clinical trials. Randomized controlled trials are indeed the gold standard for establishing causality, but Mendelian randomization offers a valuable analytic tool for assessing possible causality in observed associations when randomized controlled trials are not feasible [15]. True to their promise, Mendelian randomization studies of sleep metrics have provided important insights: In one study, abnormal sleep duration and insomnia were identified as potential causal risk factors for shortened lifespan [16, 17], and in another, insomnia was identified as a potential risk factor for incident ovarian cancer and poorer survival [18]. Mendelian randomization studies of sleep apnea have similarly suggested a potential causal relationship with the development of cardiovascular disease [19]. This line of investigation may provide further evidence clarifying whether insomnia is a greater predictor of early death than sleep apnea or COMISA.

In summary, the study by Razjouyan and colleagues yields somewhat counterintuitive findings that must be interpreted considering the many limitations of the investigators’ approach. If insomnia truly confers a greater risk of shortened lifespan than sleep apnea, or even COMISA, then there may be a compelling rationale for shifting clinical priorities and reallocating resources. Such a finding would also lend new urgency to efforts to identify the insomnia endophenotypes associated with the greatest risk and to clarify the biological pathways linking insomnia to premature mortality. More broadly, the report underscores the importance of interpreting emerging findings within the context of existing knowledge. An important challenge with rapidly growing capacity for data analytics is to present new information in a way that is both sufficiently comprehensive and appropriately contextualized, while remaining concise enough to manage the cognitive load of our times.

## Data Availability

Not applicable.

## Declarations

### Disclaimer

The author is a employee of the Veterans Health Administration. The views presented here are the author’s own and do not reflect the view of the VHA or any other agency of the US Government.
